# Glycogen Hepatopathy: A Reversible yet Relapsing Cause of Hepatitis in Type 1 Diabetics

**DOI:** 10.7759/cureus.13441

**Published:** 2021-02-19

**Authors:** Mishah Azhar, Muhammad Hammami, Ahmad Musmar, Matthew Bromer

**Affiliations:** 1 Internal Medicine, Florida Atlantic University, Boca Raton, USA; 2 Gastroenterology, Bethesda Hospital East, Boynton Beach, USA

**Keywords:** glycogenic hepatopathy, gastroenterology, diabetes type 1

## Abstract

Glycogen hepatopathy (GH), a rare glycogen storage disease caused by genetic or acquired overactivation of hepatic glycogen synthesis enzymes, can mimic non-alcoholic fatty liver disease (NAFLD). We describe a case of biopsy-proven GH in an adult with type 1 diabetes mellitus (DM).

A 33-year-old Honduran woman with a 25-year history of type 1 DM complicated by gastroparesis, multiple episodes of diabetic ketoacidosis (DKA) and hypoglycemia, and recurrent pancreatitis was referred for abnormal liver enzymes. Family history was negative for liver disease. There was no history of alcohol or recreational drug use. Patients' medications included insulin and thyroxine. Physical exam showed hepatomegaly but no stigmata of chronic liver disease. Aspartate aminotransferase (AST) and alanine aminotransferase (ALT) had ranged from 100’s to over 7000 U/L while alkaline phosphatase (ALP) was elevated to over 400 IU/L. Albumin, total bilirubin, platelets, international normalized ratio (INR), eosinophils, viral hepatitis panel, antinuclear antibody (ANA), smooth muscle antibody (Ab), anti-liver-kidney microsomal (LKM) Ab, celiac serologies, ceruloplasmin, alpha 1 antitrypsin, iron studies, and acetaminophen levels were all normal. An abdominal ultrasound showed “fatty liver” and an atrophic pancreas. CT abdomen showed hepatomegaly. The common bile duct (CBD) was found to be normal on endoscopic ultrasound (EUS) and magnetic resonance cholangiopancreatography (MRCP). A liver biopsy was pursued eventually, demonstrating glycogenotic hepatocytes.

GH is frequently misdiagnosed as NAFLD, a more common liver disease that occurs in association with diabetes While GH is known to be reversible, NAFLD has been known to progress to advanced liver disease, ranging from cirrhosis to hepatocellular carcinoma. Definite diagnosis often requires liver biopsy because of overlapping clinical and radiographical pictures. Elevation of both glucose and insulin levels in the setting of fragile DM control is thought to play a role via overstimulation of glycogen synthesis. Recommended treatment is stable “tight” glycemic control; pancreatic transplantation has resulted in sustained GH remission in two case reports. The required degree of stability and tightness of glucose control is not yet known. An increased awareness of GH is needed in an attempt to prevent delay in diagnosis, in a condition with an otherwise unknown incidence.

## Introduction

Glycogen hepatopathy (GH) was first discovered in the 1930s in children with brittle diabetes, as part of a condition known as Mauriac syndrome with these patients demonstrating growth retardation and Cushingoid features [1]. It was then shown that GH can actually be present without Mauriac syndrome and can be found in adult patients with poorly controlled type 1 diabetes. GH has since then been described as a rare condition that often goes unrecognized, not often included in the differential diagnosis in patients with elevated liver transaminases [2]. More importantly, it often gets confused with non-alcoholic fatty liver disease (NAFLD), a more common liver abnormality associated with uncontrolled diabetes mellitus (DM) [3]. GH is due to glycogen accumulation in hepatocytes and presents with massive hepatomegaly and elevated liver transaminases from acute parenchymal liver injury [1]. Other terms that have been used to describe this condition are hepatic glycogenosis, liver glycogen storage, liver glycogenosis, and DM-associated glycogen storage hepatomegaly [3]. We describe a case of GH that, similar to cases described in the current literature, not only portrays the challenge faced by physicians when diagnosing this condition, but also the importance of distinguishing this from NAFLD. Our aim, in this case, is to increase awareness of GH in an attempt to prevent delay in diagnosis, in a condition with unknown incidence.

This case report was previously presented as an abstract at the ACG 2019 Annual Scientific Meeting Abstracts; San Antonio, Texas [4].

## Case presentation

Our patient is a 33-year-old Honduran female with poorly-controlled diabetes (hemoglobin A1c of 9.5% to 11%), diagnosed at the age of 8, requiring multiple hospitalizations secondary to diabetic ketoacidosis (DKA) with findings of elevated aminotransferases [4]. Her medical history is notable for severe gastroparesis, requiring endoscopic botox injections, recurrent pancreatitis of unknown etiology, and hypothyroidism. Her surgical history was significant for cholecystectomy done in her mid-20s. In terms of her social history, she had denied any alcohol use, smoking, or usage of any recreational drugs. There was no known family history of any gastrointestinal pathology.

At the age of 25, the patient had initially presented with dyspepsia, early satiety, and diarrhea that required her to obtain an endoscopy demonstrating gastritis but with no other abnormal findings otherwise. During that time, she required a five-day hospital stay due to noted abnormal liver tests that were attributed to gastroenteritis at that time, as the patient had no history of alcohol abuse, no evidence of cholelithiasis, and a negative hepatitis panel. During her mid-20s, the patient was found to have numerous hospital admission secondary to DKA, at which time the patient was found to have erratic blood sugars (40s-500s) with an endocrinologist closely following her case. Her diabetes was being treated with both Tresiba (insulin degludec) and inhaled insulin, Afrezza. It was at this time that she was also found to have significant elevations in her liver tests, which consisted (at their highest) of an aspartate aminotransferase (AST) of 7,184 U/L (reference: 8-48 U/L), alanine aminotransferase (ALT) of 650 U/L (reference: 7-55 U/L), and alkaline phosphatase (ALP) of 443 U/L (reference: 40-129 U/L). Physical exam demonstrated hepatomegaly, which was confirmed on CT. She continued to present to the hospital numerous times with these flares, found to have fluctuations in both her blood glucose levels and aminotransferases. Other imaging, such as an abdominal ultrasound during these times had demonstrated fatty liver with an atrophic pancreas. She also had had multiple endoscopic ultrasounds and magnetic resonance cholangiopancreatography (MRCP) that demonstrated normal common bile duct (CBD). Due to this, the patient was tested for numerous other etiologies for the cause of these findings, including autoimmune hepatitis, infectious, ischemic, and drug-induced hepatitis, all being ruled out at this time. Some of these tests included a hepatitis panel, acetaminophen levels, ceruloplasmin level, alpha-1-anitrypsin level, anti-tissue transglutaminase antibody just to name a few.

**Figure 1:**
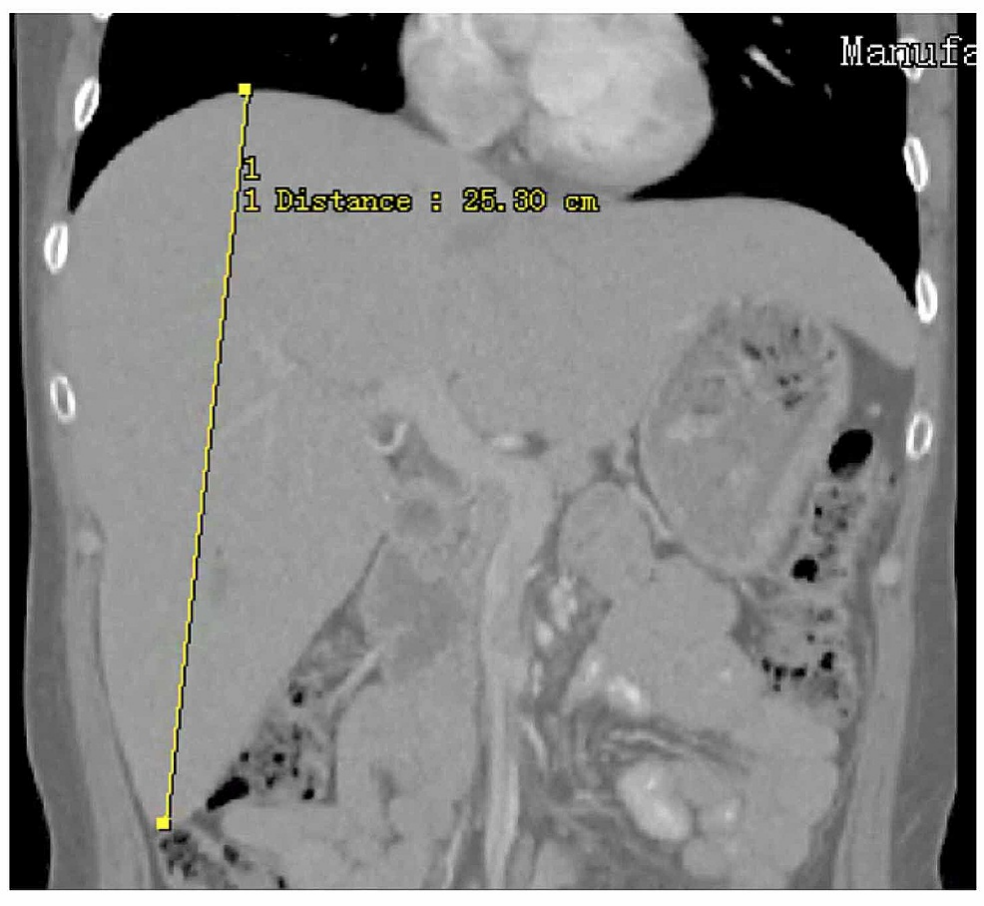
MRI of the abdomen demonstrating markedly enlarged liver during one of the patient's hospitalizations

**Figure 2:**
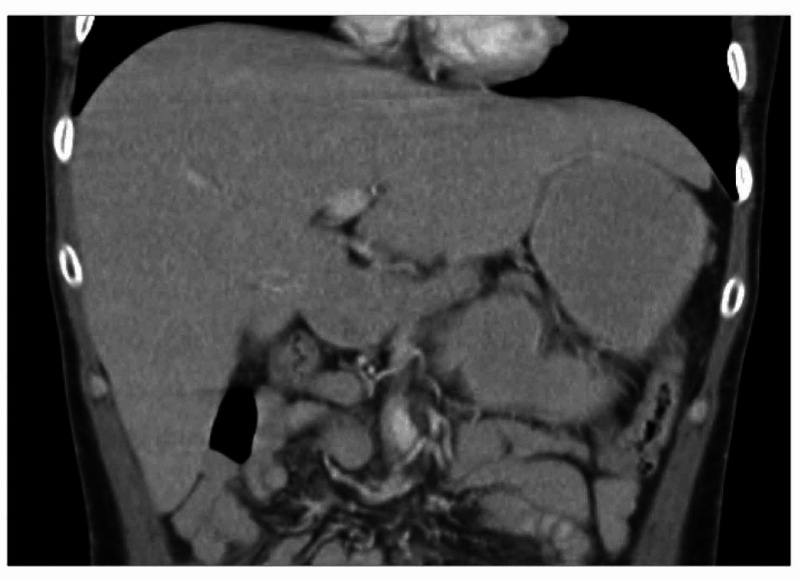
MRI of the abdomen during one of the patient's hospitalizations with improved hepatomegaly, demonstrating the reversibility of this condition

Finally, after exhausting some of these diagnostic tests, our patient was sent for a liver biopsy for further evaluation and to definitively rule out an autoimmune cause for her condition. The liver biopsy, however, demonstrated glycogenotic hepatocytes with the patient subsequently being diagnosed with GH. Subsequent hospitalizations were due to abdominal pain, nausea, and vomiting secondary to gastroparesis and chronic pancreatitis. She continued to frequently obtain endoscopic Botox injections for her gastroparesis, as well as celiac plexus block for the alleviation of her pain due to pancreatitis. Her blood glucose levels remained better controlled during these later hospitalizations, with less frequent hypoglycemic episodes, which was reflected on her liver tests that appeared mildly elevated to almost normal. This improvement was additionally portrayed on repeat imaging done during one of the patient's later hospitalizations (Figures 1-2).

## Discussion

GH is a condition that has been associated with uncontrolled type 1 DM, particularly in those with fluctuations in both blood glucose and insulin levels. It has rarely been seen in uncontrolled type 2 diabetics, and even less frequently has been associated with conditions such as dumping syndrome after gastrectomy, anorexia nervosa, use of high-dose steroids, azathioprine, and insulin overdose [5]. None of which were present in our patient. It is a condition with no known gender predominance and is seen in both children and adults [6].

The pathophysiology of GH is due to the transport of glucose into hepatocytes due to its conversion to glucose-6-phosphate via glucokinase and then subsequently being converted to glycogen via glycogen synthase [6]. Glycogen synthase is activated after dephosphorylation by phosphatase, an enzyme whose concentration and activity is dependent on insulin and the presence of glucose [6]. This is the main determinant for why both an elevation of blood glucose and insulin levels are needed for the development of GH [6]. Known exacerbating factors are frequent hypoglycemic episodes, as witnessed in our patient, that are commonly seen in type 1 diabetic patients. In fact, hypoglycemia from poor diabetes control, along with aggressive insulin therapy, is what drives the development of this condition, as seen in our patient [3]. Its rarity might be due to defects in genes coding for regulatory proteins, such as laforin, which in turn regulates the activity of glycogen synthase and/or glucose-6-phosphatase [2]. Another study demonstrated that a structural defect on the glycogen phosphorylase gene (PYGL) may be involved in glycogen accumulation [6].

Clinically, patients with GH can present with many nonspecific signs and symptoms, such as abdominal pain, nausea, and vomiting, the most common being hepatomegaly with lab work indicative of elevated transaminases. While lab results typically demonstrate elevated liver enzymes with a predominantly hepatocellular pattern, they may also have elevated alkaline phosphatase or bilirubin levels indicating cholestatic injury (albeit a much smaller component) as was the case in our patient [6]. Another important finding that may help distinguish this condition from others is the AST dominant elevation, most often seen in these patients, that was most definitely seen in our patient [7]. In a patient presenting like ours, many different diagnoses come to mind, with ischemic, infectious, and autoimmune causes being the top of the differentials. In our case, these causes were ruled out immediately with the laboratory tests that had been ordered.

Based on the above findings, it is easy to see why this condition is most frequently misdiagnosed with NAFLD, another condition associated with diabetes, but with a very different prognosis. While GH has been shown to be reversible, NAFLD has been known to progress to advanced liver disease, ranging from cirrhosis to hepatocellular carcinoma [7]. The challenge is distinguishing GH from NAFLD as both appear the same, clinically and radiographically, with a definitive diagnosis made with liver biopsy. Histologically, in GH, you will discover marked glycogen accumulation leading to pale, swollen hepatocytes on hematoxylin and eosin stain with an otherwise intact architecture and no significant fibrosis [6]. Staining with periodic acid-Schiff (PAS) demonstrates a significant amount of cytoplasmic glycogen deposits, disappearing after digestion with diastase [6]. Many other conditions may be associated with these findings, including glycogen storage disease, high-dose steroids, and medications (phenytoin); findings that can be easily excluded with a thorough history from our patients [6]. However, while abdominal ultrasound may not be helpful in distinguishing these two conditions, a CT abdomen may be able to provide some clues with the liver appearing more dense and, therefore, bright in patients with GH vs those with NAFLD [6]. Another diagnostic modality that has been described in the literature is a gradient dual-echo MRI, that may distinguish glycogen from fat and further assist in differentiating it from NAFLD, with the sole purpose of using these less invasive options as clues in diagnosing this condition earlier in the course [8].

While the exact mechanism of this condition is still being studied, the one thing we do know is that treatment consists of tighter glycemic control, with noticeable changes noted within 2 to 14 weeks [7]. Although, in our patient, and many other published cases, the glycemic control may not always correlate directly with elevation of liver tests. In fact, like our patient whose transaminases improved despite improvements in glycohemoglobin levels, there is still much to be known about the degree of improved glycemic control needed for the resolution of GH [9]. In one study, two patients treated with pancreas transplantation were noted to have a complete reversal of their GH, inducing an insulin-independent, euglycemic state [10]. This procedure was performed in those with severe secondary complications of diabetes, including kidney failure [10]. Although our patient did not have kidney failure, she did have severe gastroparesis requiring frequent endoscopic Botox injections could possibly be a candidate for pancreatic transplantation.

## Conclusions

Based on our case and review of the current literature, there is still much to be known about GH. While this condition has been around since the 1930s, there is still a paucity of knowledge regarding the biochemical defects, prevalence, characteristics, and risk factors involved. While we know this condition relies upon elevation of both glucose and insulin levels, the improvement in glycemic levels needed prior to the resolution of GH is still something that remains undetermined. We hope that by presenting this case, it allows physicians to be aware of a disease that is often confused with a more well-known condition that presents almost identically, known as NAFLD. This will hopefully lead us to further investigate the role of both the aforementioned and hopefully newer diagnostic modalities in distinguishing GH and whether this can help prevent a delay in diagnosis. More than anything, this case demonstrates the importance of keeping GH in the differential when you are investigating the cause of elevated liver enzymes, particularly in an uncontrolled type 1 diabetic.
